# Clinical outcomes in 2481 unselected real-world patients treated with a polymer-free sirolimus-eluting stent: 3 years results from the NANO multicenter Registry

**DOI:** 10.1186/s12872-021-02356-0

**Published:** 2021-11-12

**Authors:** Yi Dai, Rutao Wang, Fengying Chen, Yaojun Zhang, Yi Liu, He Huang, Ping Yang, Ruining Zhang, Bo Zheng, Chao Gao, Yundai Chen, Ling Tao

**Affiliations:** 1 Medical School of Nankai University, Tianjin, China; 2 Department of Cardiology, The First Affiliated Hospital of The Fourth Military Medical University, Xi’an, Shaanxi China; 3 Department of Cardiology, The Affiliated Hospital of Inner Mongolia Medical University, Hohhot, China; 4 Department of Cardiology, Xuzhou Third People’s Hospital, Xuzhou Medical University, Xuzhou, China; 5 Department of Cardiology, Xiangtan Central Hospital, Xiangtan, China; 6 Department of Cardiology, China Japan Union Hospital of Jilin University, Changchun, China; 7 Department of Cardiology, The Chinese PLA General Hospital, 28 Fu xing road, Beijing, 100853 China; 8 Xijing Hospital, 127 Changle west road, Xi’an, 710032 China

**Keywords:** Real-world population, Percutaneous coronary interventions, Polymer-free drug-eluting stent, Target lesion failure

## Abstract

**Objectives:**

To evaluate the 3-year clinical outcomes of a polymer-free sirolimus-eluting, Nano plus stent for the treatment of coronary artery disease in the NANO multicenter Registry.

**Background:**

The long-term clinical data evaluating the safety and efficacy of the novel polymer-free sirolimus-eluting Nano plus stent (Lepu Medical, Beijing, China) is limited.

**Methods:**

The NANO all-comers Registry trial was a prospective, multicenter clinical registry conducted in 26 centers in China between August 2016 and January 2017. A total of 2481 consecutive patients were exclusively treated with the Nano plus stent. The primary clinical endpoint, target lesion failure (TLF, defined as cardiac death, target vessel nonfatal myocardial infarction, and clinically driven target lesion revascularization [CD-TLR]), was analyzed at 3 years.

**Results:**

At 3 years, 2295 patients (92.5%) were followed. The incidence of TLF was 6.8% (168/2481). The rate of cardiac death was 3.8% (94/2481), target vessel nonfatal myocardial infarction 0.7% (18/2481), and CD-TLR 2.9% (68/2481). The rate of definite/probable stent thrombosis was 0.5% (13/2481). The risk factors of diabetes mellitus, acute myocardial infarction, age, chronic renal failure, in-stent restenosis, chronic total occlusion, and left ventricular ejection fraction < 40% were the independent predictors of 3-year TLF.

**Conclusions:**

At three years, the rate of TLF was relatively low in patients treated with the polymer-free Nano plus stent. The polymer-free Nano plus stent showed a favorable safety and efficacy profile in real-world patients.

*Clinical trial registration* URL: https://www.clinicaltrials.gov/. Unique identifier: NCT02929030.

## Introduction

Percutaneous coronary interventions (PCI) with drug-eluting stents (DES) are currently the most common revascularization treatment strategy for coronary artery disease worldwide. DES has dramatically improved clinical outcomes compared to the bare metal stent (BMS) [1]; however, current DES systems always need relatively long (> 6 months) dual antiplatelet therapy (DAPT) [2], which confined their usage on a significant proportion of patients with adherence restraints, such as those at high bleeding risk [3].

The durable polymer has been demonstrated to be associated with vessel wall inflammation and contributes to delay arterial healing, which could lead to late thrombotic risk [4, 5]. Polymer-free coating technologies have then emerged. Polymer-free DESs aim to prevent adverse events caused by hypersensitivity reactions and chronic inflammation to polymer [6, 7]. In patients at high bleeding risk, polymer-free DES was found to be superior to BMS when used with a 1-month course of DAPT [8].

The Nano plus stent is a novel polymer-free stent with nano-sized pores as drug carriers that contain the antiproliferative drug sirolimus and is one of the most widely used DESs in China. Nano plus stent has an improved uniform distribution on the adluminal stent surface than microporous or textured rough surface stents. Nano plus stent has been demonstrated to have comparable safety and efficacy to durable polymer DES for treating de novo coronary artery lesions in a selected randomized controlled trial population [9]. However, the efficacy and safety of the Nano plus stent in real-world practice remained scarce. Previously, we reported the 1-year results of the NANO Registry, showing that the clinical outcome of the Nano plus stent was associated with a low rate of TLF [10]. The current study reports the 3-year outcomes of the NANO all-comers Registry.

## Methods

### Study design and population

The NANO all-comers Registry trial (NCT02929030) was a prospective, multicenter trial conducted in 26 centers across China between August 2016 and January 2017 with a single arm design. A total of 2481 consecutive patients with symptomatic coronary artery disease scheduled for PCI were enrolled, with no specific inclusion or exclusion criteria [10]. The NANO Registry planned to follow the patients up to 5 years. Patients were contacted at 30 days, 180 days, and 1 year by telephone or scheduled outpatient clinic visit. After 1 year, telephone contact was conducted annually to assess the clinical status and adverse events. In the NANO Registry, each patient provided at least two telephone numbers when he/she participated in the study. If investigators could not reach patients at follow-up, the protocol mandated all possible efforts to be made to trace the patients. Family members or referring cardiologists were contacted if necessary.

The trial was performed in accordance with the Declaration of Helsinki and was approved by the ethics committees of the Xijing Hospital. All the patients signed the written informed consent prior to participation in the trial. Clinical outcomes were adjudicated by an independent clinical event committee, and three CEC members reviewed all the available cine films and adjudicated the events for each event.

### Outcomes

The primary clinical endpoint was target lesion failure (TLF), defined as cardiac death, target vessel nonfatal myocardial infarction (MI), and clinically driven target lesion revascularization (CD-TLR). The safety endpoint was definite and/or probable stent thrombosis (ST). MI was defined according to the third universal definition [11]. Repeat revascularization was defined as any repeat revascularization by PCI or coronary artery bypass graft. ST was defined according to the Academic Research Consortium criteria [12].

### Study device

The Nano plus stent is a novel polymer-free sirolimus-eluting stent (Lepu Medical, Beijing, China) with the nanoporous stent surface technology used to carry drug and control drug release. The Nano plus stent system is based on a 316L stainless steel platform and has a high-pressure delivery system with a semi-compliant rapid exchange balloon catheter. The delivery system presents a crossing profile of 0.9–1.2 mm with two radiopaque markers at the ends of the balloon to facilitate correct stent placement. The two ends of the stent have a sinusoidal curve shape, while the center of the stent is composed of a specialized cyclic structure that aligns into a helix. Nano-sized pores (mean pore diameter: 400 nm, 1/800 of the stent thickness) are uniformly distributed on the abluminal stent surface.

### Study procedures and medications

In the NANO Registry, PCI was performed per the standard of practice of each participating center [13–15]. We recommend that all patients were pretreated with aspirin and a P2Y_12_ inhibitor (clopidogrel or ticagrelor) according to the standard of care and provided DAPT for ≥ 6 months (stable patients) or ≥ 12 months (acute coronary syndrome) according to the guidelines [13–15]. The continuation of DAPT beyond the duration of the recommended guidelines was performed at the physician's discretion. Additional medications for secondary prevention, including statins, beta-blockers, and angiotensin-converting enzyme inhibitors, were prescribed according to the guidelines [13–15].

### Statistical analysis

Continuous variables are presented as mean ± standard deviation. Categorical data are expressed as percentages. Cumulative event curves were generated using the Kaplan–Meier method. A multivariable Cox proportional hazards model was used to identify the independent predictors of the 3-year TLF. Baseline clinical and procedural variables that were considered clinically relevant or that showed a univariate relationship with TLF (*p* < 0.10) were entered into the multivariate Cox proportional hazards model. All statistical analyses were performed using the SAS 9.1 software package (SAS Institute, Cary, NC). All tests were 2-sided, and a *p* value < 0.05 was considered statistically significant.

## Results

### Baseline demographics and clinical characteristics

Patient baseline and lesion characteristics are reported previously and also in Tables 1 and 2 [10]. The mean age of the patients was 62.8 ± 10.1 years. 40.2% of patients presented acute myocardial infarction (AMI), and 22.8% of patients had diabetes mellitus. 11.6% of patients had multiple vessel PCI, and 63.9% of lesions were American College of Cardiology/American Heart Association (ACC/AHA) type B2 or C lesions, including 17.0% ultra-long lesions (lesion length ≥ 40 mm), 14.5% chronic total occlusions (CTO), 11.7% bifurcations, 5.8% severe calcifications, 2.7% severe tortuosity, and 4.1% referenced vessel diameter < 2.5 mm.

**Table 1:** Baseline demographics and clinical characteristics

Clinical characteristics	N = 2481
Age, years (mean ± SD)	62.76 ± 10.07
Sex (male)	1763 (71.1%)
DM	564 (22.8%)
Insulin treatment for DM	158 (28.0%)
Hypertension	1388 (56.0%)
Chronic renal failure	41(1.7%)
Hypercholesterolemia	1046 (42.2%)
Previous MI	320 (12.9%)
Previous PCI	278 (11.2%)
Previous CABG	17 (0.7%)
Current smoker	56.82 ± 9.25
*Clinical presentation*	
Silent ischemia	54 (2.2%)
Stable angina	170 (6.7%)
Unstable angina	1298 (52.4%)
Non-ST-elevation MI	324 (13.1%)
ST-elevation MI	672 (27.1%)

*DM* diabetes mellitus, *MI* myocardial infarction, *PCI* percutaneous coronary intervention, *CABG* coronary artery bypass graft

**Table 2:** Lesion and procedural characteristics

Variable	N = 2904
*Target vessel*	
LMCA	96 (3.3%)
LAD	1278 (44.0%)
LCX	586 (20.2%)
*TIMI flow grade preprocedure*	
0	599 (20.7%)
1	206 (7.1%)
2	218 (7.5%)
3	1871 (64.7%)
Thrombus present	474 (19.1%)
Thrombus aspiration	114 (5.7%)
*Lesion complexity*	
AHA/ACC classification	
A	428 (15.0%)
B1	428 (15.0%)
B2	506 (17.8%)
C	1315 (46.1%)
In-stent restenosis, n (%)	57 (2.0%)
Calcified lesion	169 (5.8%)
Bifurcated lesions	340 (11.7%)
Chronic total occlusion	422 (14.5%)
Severe tortuosity	79 (2.7%)
Lesion length ≥ 40 mm	460 (17.0%)
RVD < 2.5 mm	115 (4.1%)
Total stent diameter	3.11 ± 1.34
Total stent length, mm	25.99 ± 8.59
Post dilatation	2050 (70.6%)
Average maximal pressure (atm)	17.45 ± 3.82
Multiple vessel PCI	287 (11.6%)
Number of stents per lesion	1.33 ± 0.60
Number of stents per patient	1.56 ± 0.79
Device success	2385 (96.1%)
Procedural success	2368 (95.4%)
*P2Y12 at discharge*	
Clopidogrel	1785 (71.9%)
Ticagrelor	696 (28.1%)

Values are expressed as mean ± SD or n (%)

*ACC* American College of Cardiology, *AHA* American Heart Association, *LAD* left anterior descending artery, *LCX* left circumflex artery, *LMCA* left main coronary artery, *PCI* percutaneous coronary intervention, *RCA* right coronary artery, *TIMI* thrombolysis in myocardial infarction, *RVD* referenced vessel diameter

### Clinical outcomes up to 3 years follow-up

**Fig. 1:**
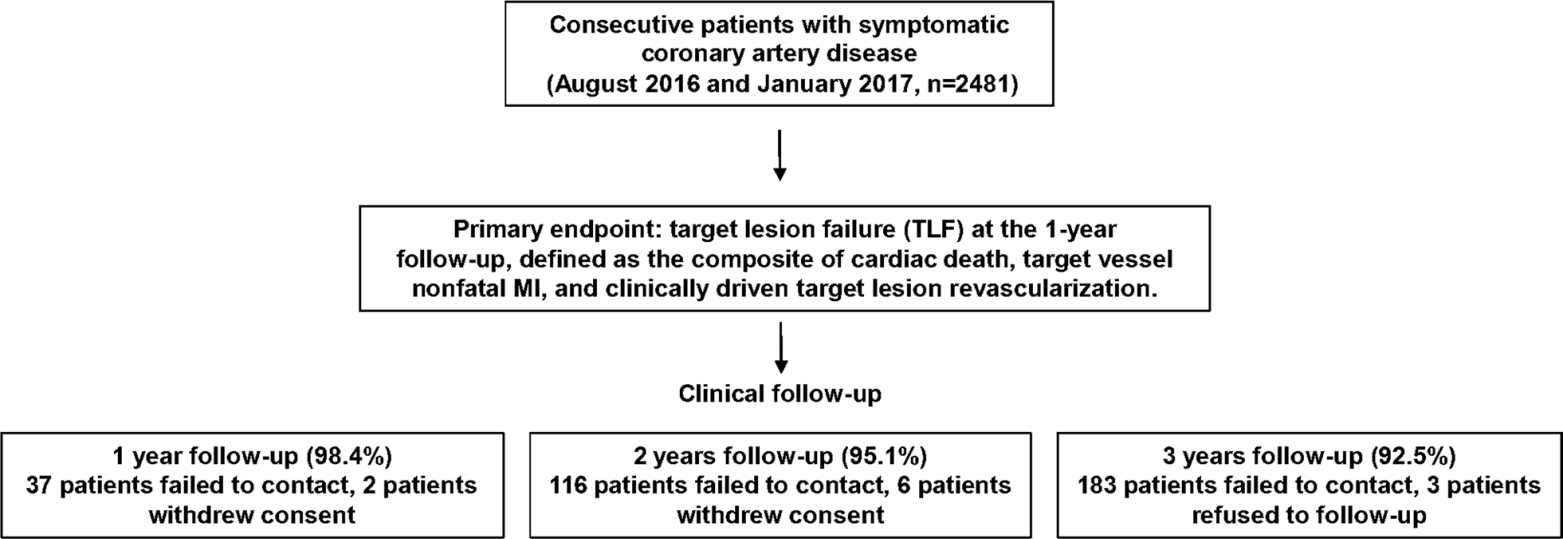
Study flow chart

**Fig. 2:**
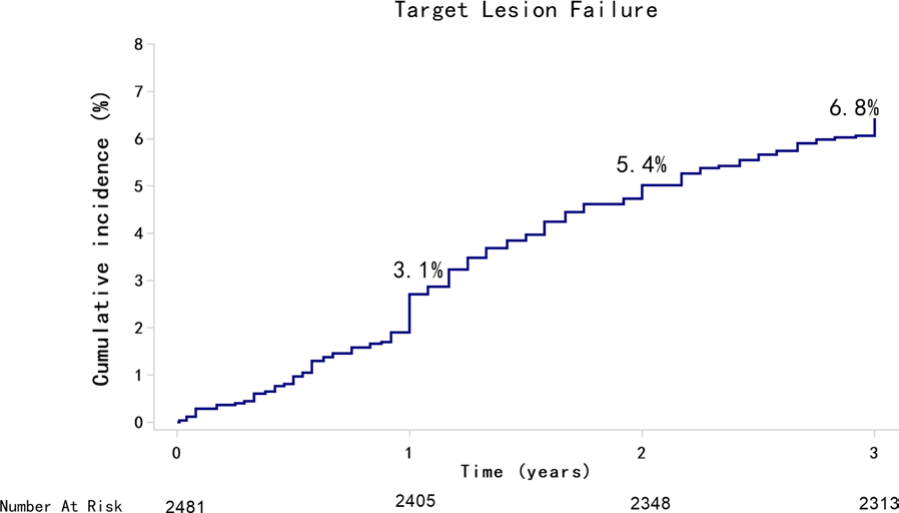
Cumulative incidence of target lesion failure up to 3 years

**Fig. 3:**
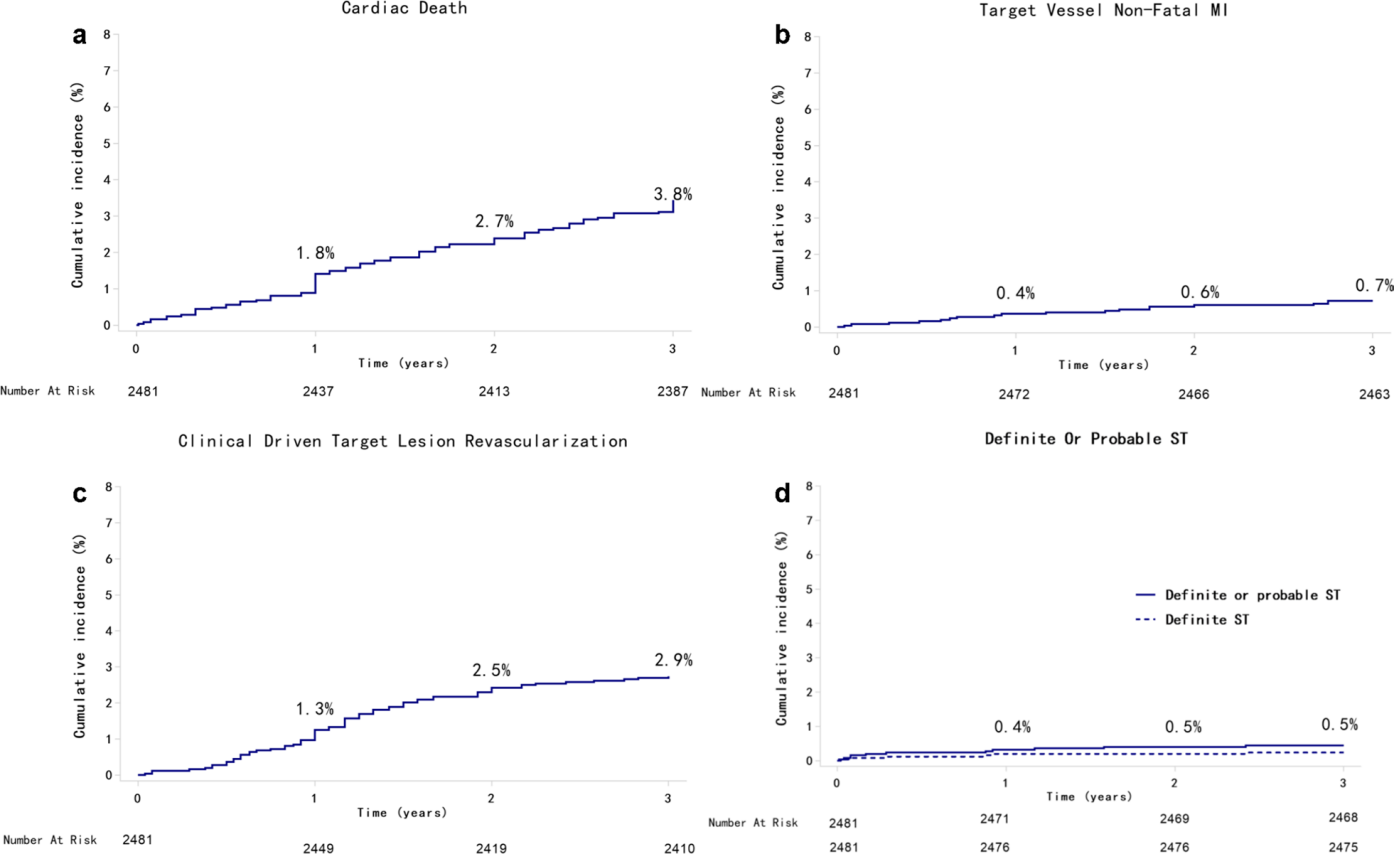
Cumulative incidence of cardiac death (**a**), target vessel nonfatal MI (**b**), clinical driven TLR (**c**), and ST (**d**) up to 3 years. TLR, target lesion revascularization; MI, myocardial infarction; ST, stent thrombosis

A total of 2295 patients (92.5%) were followed up for 3 years, and 186 patients were lost to follow-up (Fig. 1). The cumulative rate of TLF at 3 years was 6.8% (n = 168) among all patients (Fig. 2), with cardiac death occurred in 3.8% (n = 94) (Fig. 3a), target vessel nonfatal MI in 0.7% (n = 18) (Fig. 3b), and CD-TLR in 2.9% (n = 68) (Fig. 3c) of patients (Table 3). At 3 years, the rate of definite or probable ST was 0.5% (n = 13) (Fig. 3d, Table 3). The rates of clinical outcomes within 1 year, 1–2 years, and 2–3 years are shown in Table 3.

**Table 3:** Clinical outcomes up to 3 years follow-up

Endpoint	0–1 year n (%)	1–2 year n (%)	2–3 year n (%)	0–3 years n (%)
Target lesion failure	76 (3.1%)	57 (2.3%)	35 (1.4%)	168 (6.8%)
Cardiac death	44 (1.8%)	24 (1.0%)	26 (1.0%)	94 (3.8%)
Target non-fatal vessel MI	9 (0.4%)	6 (0.2%)	3 (0.1%)	18 (0.7%)
Clinically driven TLR	34 (1.3%)	30 (1.2%)	9 (0.4%)	73 (2.9%)
Definite stent thrombosis	5 (0.2%)	0 (0%)	1 (0.04%)	6 (0.2%)
Definite/Probable stent thrombosis	10 (0.4%)	2 (0.1%)	1 (0.04%)	13 (0.5%)

### Predictors of TLF at 3 years follow-up

Multivariate analyses of TLF at 3 years using the Cox proportional hazard model showed that the independent predictors of 3-year TLF included diabetes mellitus, AMI, age, chronic renal failure, in-stent restenosis, CTO, and left ventricular ejection fraction < 40% (Table 4).

**Table 4:** Predictors of TLF at 3 years follow-up

Predictors	Univariate		Multivariate	
	HR (95% CI)	*p* value	HR (95% CI)	*p* value
Age	1.04 (1.02–1.06)	< 0.01	1.03 (1.01–1.05)	0.003
DM	1.79 (1.30–2.47)	< 0.01	1.78 (1.20–2.56)	0.003
AMI	1.46 (1.08–1.97)	0.015	1.47 (1.02–2.12)	0.038
Chronic renal failure	2.93 (1.21–7.15)	0.018	2.80 (1.12–7.01)	0.028
LVEF < 40%	3.70 (2.32–5.92)	< 0.01	3.02 (1.88–4.87)	< 0.01
ISR	2.48 (1.22–5.05)	0.012	2.32 (1.12–4.81)	0.024
CTO	1.74 (1.22–2.47)	< 0.01	1.53 (1.003–2.33)	0.048

*AMI* acute myocardial infarction, *CTO* chronic total occlusion, *DM* diabetes mellitus, *ISR* n-stent restenosis, *LVEF* left ventricular ejection fraction

## Discussion

Results of the 3 years analysis of the NANO all-comers Registry show that patients treated with polymer-free Nano plus stent had a relatively low rate of TLF and definite or probable ST, suggesting the polymer-free Nano plus stent was safe and effective in a real-world population.

Stent design, antiproliferative drug, and the presence and type of polymer are the key factors of a DES platform relate to its clinical efficacy. The polymer of DES, which can facilitate loading and controlling the release of antiproliferative drugs, was suspected to be related to inflammatory responses and delayed arterial healing [4]. Polymer-free DESs were initially designed out of hopes that, without the polymer, the risk of polymer-related inflammation and late thrombotic events would be decreased.

However, the safety and efficacy of the polymer-free DES, as compared to durable polymer DES or bioresorbable polymer DES, is still under debate. The SORT OUT IX found that the polymer-free BioFreedom stent did not meet the criteria of non-inferiority regarding MACE (major adverse cardiac events) at 12 months when compared with the ultrathin strut BP sirolimus-eluting Orsiro stent, and the BioFreedom stent had a higher incidence of TLR [16]. In contrast to these findings, most studies demonstrated that compared to durable polymer DES, polymer-free DES has a non-inferior efficacy profile either in a selected population [17, 18], or in a real-world clinical setting [19, 20]-even follow up to 10 years [21].

Several features of the BioFreedom stent should be considered. It has been demonstrated that DES efficacy is closely associated with the release kinetics of the antiproliferative drug in the first 30 days; however, around 90% of biolimus A9 is released from the BioFreedom stent within 48 h of implantation, and the relatively fast drug release may contribute to less efficacy on inhibiting neointimal hyperplasia [16]. Strut thickness is associated with in-stent restenosis [22]. The BioFreedom stent is with a strut thickness of 112 µm, which is thicker than most other newer-generation DES (60–90 µm).

We previously reported that the polymer-free Nano plus stent showed similar safety and efficacy compared with the durable polymer sirolimus-eluting stent in terms of angiographic outcomes at 9 months and clinical outcomes at 2 years [9]. In the current analysis, we showed that using polymer-free Nano plus stent for the treatment of relatively complex lesions in the unselected population is associated with a low and acceptable rate of 3-year TLF. Compared to the BioFreedom stent, the Nano plus stent has a relatively thin strut (91 μm), and 85% of the sirolimus is released within 30 days [9]. However, since there is no data comparing BioFreedom and Nano plus stent, the above-mentioned potential advantage of Nano plus stent is only theoretical and hypothetical.

Compared to durable polymer DES, another benefit of polymer-free DES is its ability to allow a shorter DAPT course after stent implantation. The LEADERS FREE trial showed that polymer-free DES (BioFreedom) has an efficacy and safety advantage over BMS at 1 year in patients at high bleeding risk treated with 1-month DAPT [8]. The benefit of polymer-free DES over BMS was maintained for up to 2 years [23]. The LEADERS FREE II trial reproduced the results of LEADERS FREE in an independent, predominantly North American cohort of high bleeding risk patients [24]. Notwithstanding, this advantage seems to be recently challenged. The ONYX ONE trial observed that among patients at high bleeding risk who received 1 month of DAPT after PCI, durable polymer DES (Resolute Onyx) was non-inferior to polymer-free DES (BioFreedom) in terms of safety and effectiveness composite outcomes [25]. The efficacy of Resolute Onyx and Nano plus stent warrant to be compared in future studies enrolling high bleeding risk population.

Stent failure remains to occur, which may lead to adverse cardiac events, despite the improvement of the contemporary DES. Identifying the related factors that may predict TLF was of paramount importance. However, predictors of TLF vary in the different postprocedural periods [26]. Previously, we identified that diabetes mellitus, AMI, left ventricular ejection fraction < 40%, and long lesions (> 40 mm) independently predicted 1-year TLF. At 3 years, the independent predictors of TLF included diabetes mellitus, AMI, age, chronic renal failure, in-stent restenosis, CTO, and left ventricular ejection fraction < 40%, the predictors identified in the present analysis are highly consistent with previous studies [26–28]. Identifying and intensively managing these predictors may help to reduce the rate of TLF and improve the long-term clinical outcomes.

### Limitations

The present study was a single arm, non-randomized study with inherent limitations. However, the current analysis highlights the safety and efficacy of Nano plus stent in an unselected population in a real-world setting. Although the results of the analysis showed that polymer-free Nano plus stent has a favorable safety and efficacy profile, head-to-head comparisons with the newer generation of DES are needed in future studies. In addition, the follow-up rate was relatively low in the present study, which could partially contribute to the low events rate.

## Conclusion

At 3 years follow-up, TLF was relatively low in patients treated with polymer-free Nano plus stents in the multicenter NANO Registry trial. The Nano plus stents showed promising safety and efficacy in real-world patients, although longer follow-up is needed for further evaluation.

## Data Availability

The datasets generated and/or analyzed during the current study are not publicly available but are available from the corresponding author on reasonable request.
